# The Relationship between Benthic Macroinvertebrate Assemblages and Water Quality Parameters in the Sanyati Basin, Lake Kariba, Zimbabwe

**DOI:** 10.1155/2022/5800286

**Published:** 2022-05-31

**Authors:** Peter Makumbe, Artwell Kanda, Tambudzai Chinjani

**Affiliations:** ^1^Shangani Ranch, P.O. Box 24, Shangani, Zimbabwe; ^2^Department of Environmental Science, Bindura University of Science Education, P. Bag 1020, Bindura, Zimbabwe

## Abstract

Biological monitoring of reservoirs is important in assessing aquatic health. This study aimed at assessing the structure of benthic macroinvertebrate communities in relation to physicochemical parameters along Sanyati basin shoreline in Lake Kariba, Zimbabwe. Six sites (S1 to S6) characterized by various human disturbances were sampled for physicochemical parameters and benthic macroinvertebrates from January to March 2018. We computed macroinvertebrate metrics and classified them into functional feeding groups (FFGs). A canonical correspondence analysis (CCA) triplot was constructed to assess species-physicochemical relations. Significant differences across the sampling sites were observed for pH, electrical conductivity, turbidity, nitrate-nitrogen (NO_3_-N), ammonium nitrogen (NH_4_-N), total nitrogen (TN), phosphate-phosphorus (PO_4_-P), total phosphates (TP), and dissolved oxygen (DO). The results from CCA highlighted that S1 was generally associated with high pH, NH_4_-N, and TN, and Oligochaeta, Syrphidae, and Hydrophilidae families. The highest percentage of EPT taxa (39.83%) was recorded at S6, while the lowest was recorded at S1. The taxa were made up of 50% predators, 26% collector-gatherers, 6% scrappers, 6% shredders, and 3% collector-filters with 3 taxa (Chironomidae, Hydropsychidae, and Leptoceridae) having more than two FFGs. Site S1 had a significantly higher mean abundance of collector-gatherers than the other sites. A high correlation between water parameters and SASS and ASPT scores was observed indicating their ability to detect environmental changes. These findings suggest that macroinvertebrate communities are good candidates for delineating the effects of industrial pollution on water quality.

## 1. Introduction

Lakes and reservoirs are critical components of freshwater ecosystems and are an important habitat for aquatic species [1, 2]. Rapid industrial and urban development has, however, compromised the integrity of many reservoirs in developing nations due to pollution from industrial and domestic use [3]. Pollutants such as nitrogen and phosphorus from such spillages may cause increased aquatic biological productivity, resulting in low dissolved oxygen (DO) and eutrophication of lakes and reservoirs and other such standing waters [4, 5]. Eutrophication in turn reduces habitat heterogeneity, thus directly or indirectly affecting aquatic life [6, 7] and reducing the health status of aquatic ecosystems.

Water quality and biological monitoring of lakes and reservoirs are critical given the increase in anthropogenic disturbances on those water bodies [5, 8]. Macroinvertebrates are important indicators of water pollution caused by physical and chemical alteration of lake water and the surrounding habitat [9, 10]. Due to their limited mobility and near sedentary lifestyle, macroinvertebrates can reflect conditions that are not present at the time of sampling [11], thus becoming effective indicators of current and long-term water quality. This is because macroinvertebrates respond to changes well before the manifestation of a problem [12]. As such understanding macroinvertebrate-environment relations is important in understanding the integrity of aquatic communities, especially within the context of habitat degradation.

The diversity and composition of macroinvertebrates are important themes in aquatic ecology as they can be used to interpret the long-term effects of water pollution. The response of macroinvertebrates to organic loading has been documented in rivers and streams [10, 13, 14], and their use as water quality indicators in those water bodies is acceptable [15, 16]. Using macroinvertebrates as indicators of water quality presents several advantages because they have narrow ecological requirements and great diversity of form and habit, making them useful bioindicators of aquatic ecological balance [14]. The rationale for the use of ecosystem health macroinvertebrate metrics is based on the observation that some taxa, especially the Ephemeroptera, Plecoptera, and Trichoptera (EPT), are sensitive to pollutants, while others such as Chironomidae and Oligochaeta are more tolerant [13, 17]. Despite this general observation, macroinvertebrate communities may respond differently to chemical pollution in terms of both structure and function [18], making it important to study their specific response in different aquatic communities.

Knowing the long-term effects of water pollution in the face of environmental perturbation is important, and the use of benthic macroinvertebrates can be useful in water quality monitoring. Several studies on water quality assessments in Zimbabwe have focused on sampling physicochemical parameters [19–21]. Although physicochemical analyses are important in assessing water quality, they do not reflect fluxes of effluent discharges in Zimbabwean water bodies as sampling is periodic and sporadic [22]. This means the use of a combination of physicochemical parameters and macroinvertebrates can capture the whole spectrum of aquatic stressors.

This study aimed at assessing the structure of benthic macroinvertebrates communities at the family level in relation to physicochemical parameters that characterize the shoreline of the Sanyati basin in Lake Kariba, Zimbabwe. The following predictions were tested: (1) physicochemical parameters and water quality metrics (i.e., South African scoring system—SASS and average score per taxon—ASPT) would differ between areas that have high human disturbance and those with low disturbance depending on the pollution stressor; (2) physicochemical parameters would affect macroinvertebrate family structure and functional composition within the study sites, due to the known anthropogenic activities along the shore; and (3) indicator taxa, that is, Ephemeroptera, Plecoptera, and Trichoptera (EPT), would be associated with areas of low pollution levels and Diptera would be associated with areas of high levels of pollution.

## 2. Materials and Methods

### 2.1. Study Area and Study Design

Lake Kariba (16.9557°S, 27.9718°E), located to the northwest of Zimbabwe along the border between Zimbabwe and Zambia, is the world's largest man-made lake and reservoir by volume at 157 km^3^ [23]. It has a catchment area of 1 193 500 km^2^ and a basin area of 687 049 km^2^ [23]. The lake has a surface area of 4 364 km^2^ at the normal operation level of 484 m a.s.l, a length of 276 km, an average width of 19 km, and an average depth of 29 m [24]. The lake is warm, nutrient-poor, and monomictic [25, 26]. It is divided into five major hydrological basins: Mlibizi (Basin 1), Binga (Basin 2), Sengwa (Basin 3), Bumi (Basin 4), and Sanyati (Basin 5). The study was conducted within the Sanyati basin, which is the most northern of the lake's five basins. The basin receives inflows from the Sanyati, Charara, Gache gache, and Nyaodza rivers. The basin holds about 25.8% of the lake's volume and covers about 22.6% of the lake's surface area [27]. The Sanyati river, which is the major river draining into the basin, drains a large part of Gokwe's agricultural land into the basin [28]. This basin is the closest to Kariba town.

**Figure 1:**
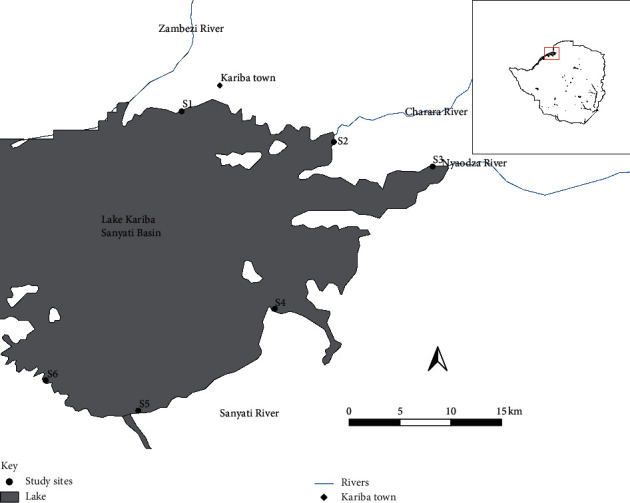
Map of Sanyati basin, Lake Kariba, showing the six (S1-S6) sampling sites between January and March 2018 (Inset: map of Zimbabwe).

For this study, six sites were sampled along the shoreline in the littoral zones of the Sanyati basin (Figure 1). Five sites were chosen to cover a spectrum of activities along the shore near Kariba town, while one site (adjacent to Matusadona National Park) was taken as a reference site as it is located far away from human habitation and previous results showed the area has low disturbance [24]. A summary of the study sites is shown in Table 1.

### 2.2. Field Sampling

Sampling at the six sites was performed twice every month for three months (January–March 2018). Five random water samples were collected at each site along the shoreline. Samples were collected at 20–30 cm water depth using one-litre sterile, acid-cleaned, polyethylene bottles [29] and preserved on ice before laboratory analysis. Water samples were collected before macroinvertebrates to prevent contamination. The determination of pH, electrical conductivity (EC), temperature, and dissolved oxygen (DO) were performed in *situ* with a calibrated digital HACH HQ30D multimeter (HACH, Loveland, CO, USA), while turbidity was determined using a portable multiparameter probe PC Testr 35 (Eutech/Oakton Instruments, Singapore). In the laboratory, nitrate-nitrogen (NO_3_-N), ammonium nitrogen (NH_4_-N), total nitrogen (TN), phosphate-phosphorus (PO_4_-P), and total phosphates (TP) were determined using a Hach DR/2010 spectrophotometer (Hach Company, 1996–2000) at the University of Zimbabwe Lake Kariba Research Station laboratory using standard spectrophotometric methods. Samples for dissolved nutrient analysis were filtered through GF/C filter paper and kept at 4°C before analysis. Analyses were performed within 5 hours of sample collection.

Macroinvertebrates were collected where water samples had been collected. At each site, macroinvertebrates were collected using a kick net (500 *µ*m mesh *D*-frame, dimensions 30 cm × 30 cm) for all the available habitat types such as macrophytes and bedrock [30]. Sampling at each site lasted for five minutes along a demarcated 10 m transect. Large debris and aquatic plants were washed and observed of any organisms. Macroinvertebrates were then put into white trays, picked, collected into jars, and preserved in 70% ethanol before sorting and laboratory identification under a Nikon dissecting microscope. All macroinvertebrates were identified to family taxonomic level using standard keys and a field guide by Gerber and Gabriel [31] and Picker et al. [32]. The abundance of invertebrates at each site was recorded.

Community composition and diversity among the six sites were assessed using the following macroinvertebrate community measures: % Diptera and % EPT commonly used to assess macroinvertebrate assemblages. Macroinvertebrates were further sorted into five functional feeding groups (FFGs): collector-filterer, collector-gatherer, predators, scrappers, and shredders [30, 33]. Benthic macroinvertebrates with more than one FFG and those FFGs represented by one family were excluded from analyses. Changes in community structure at the family level among the six sites were further assessed using five community metrics: taxa richness, Margalef richness (Dmg), Simpson's dominance (*D*), Shannon–Wiener diversity index (*H*), and Pielou evenness index (*J*). The community metrics were calculated in Paleontological Statistics (PAST) version 3.02 [34].

South African Scoring System version 5 (SASS5) and Average Score per Taxon (ASPT) have been used extensively for biological monitoring and adopted in Zimbabwe to assess water quality and aquatic health [22]. The SASS5 scores were calculated by summing up the quality scores of all the families present at a given site, irrespective of abundance [35]. The ASPT was calculated for each site following the SASS protocol by dividing the SASS score by the number of taxa [35]. The SASS and ASPT scores were used to measure site condition as follows: excellent (SASS score > 100 and ASPT score > 7, good (80–100 and 5–7), fair (60–80 and 3–5), poor (40–60 and 2–3), and very poor (<40 an*d*<2) [36].

### 2.3. Data Analyses

Mean values of physicochemical water quality parameters and macroinvertebrate metrics were compared among the six sites using a Kruskal–Wallis test [37]. Where significance occurred, means were separated by a post hoc Mann–Whitney *U* test. The test is justified because the data were not normally distributed after testing with a Kolmogorov–Smirnov test.

Due to the non-normality of the data, a Spearman's correlation analysis was used to examine the relationship between physicochemical parameters (temperature, pH, EC, turbidity, DO, NH_4_-N, NO_3_-N, TN, PO_4_-P, and TP) and macroinvertebrate metrics. The degree of association among the physicochemical parameters was determined by the correlation coefficient (*r*) and the level of significance (*p*).

All sites were ordinated in a canonical correspondence analysis (CCA), which combines taxa-site data and environmental-site data in one algorithm [38]. The CCA was performed to explain the relationship between the macroinvertebrate community assemblage and the measured physicochemical parameters to determine important parameters responsible for the observed spatial distribution of macroinvertebrate families. We first performed a background check using an unconstrained detrended correspondence analysis (DCA), which is a unimodal method. Since the gradient length was greater than 3.7 standard deviation units (4.1 in our results), we then employed a CCA ordination method [39]. Before ordination, abundance data were square root-transformed for a better fit. In the triplot, taxonomic and site data were produced as points, while environmental data were plotted as vectors. A Monte Carlo permutation test was performed to assess the statistical significance of taxa-environmental parameters for the two canonical axes [40], which were used to plot the CCA triplot. Ordination was carried out in the package Canoco for Windows 4.5 [41].

## 3. Results

### 3.1. Physicochemical Water Quality Parameters

Significant differences (Kruskal–Wallis, *p* < 0.05) across the sampling sites were observed for all water parameters (i.e., pH, EC, turbidity, and DO) except for temperature (Table 2). The lowest pH values were recorded at S6 (7.3 ± 0.05) and the highest at S2 (8.2 ± 0.07). The highest values of EC were recorded at S1 (125 ± 1.14 *µ*Scm^−1^), while the lowest value was recorded at S6 (average 93.44 ± 0.62 *µ*Scm^−1^). Turbidity was the highest at S4 (29.9 ± 0.85 NTU) and the lowest at S6 (8.9 ± 0.86 NTU). DO illustrated low concentrations at S1 (3.3 ± 0.31 mg/L) and the highest at S6 (9.4 ± 0.45 mg/L). Nutrients were found to vary significantly (Kruskal–Wallis, *p* < 0.05) among the six sampling sites with S1 having the highest concentration of NH_4_-N (0.15 ± 0.01 mg/L), TN (0.92 ± 0.06 mg/L), PO4-P (29.1 ± 0.49 *µ*/L), and TP (0.15 ± 0.004 mg/L). S6 recorded the lowest concentration of all nutrients (Table 2).

### 3.2. The Relationship between Benthic Macroinvertebrates and Physicochemical Water Quality Parameters

**Figure 2:**
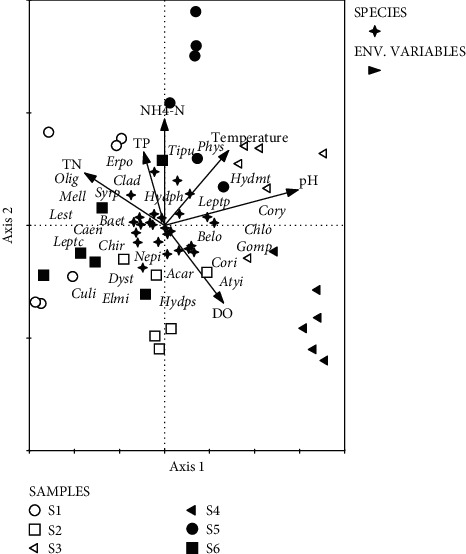
Canonical correspondence analysis (CCA) triplot for the relationship between macroinvertebrates assemblage and physicochemical variables at the six sampling sites in Sanyati basin, Lake Kariba, between January and March 2018.

CCA axes 1 and 2 were significant (Monte Carlo test, *p* < 0.05) with the eigenvalue for axis 1 and axis 2 being 0.16 and 0.06, respectively. The relative magnitude of the eigenvalues of each axis indicates the relative importance of each axis. *Axis* 1 explained 40.4%, while axis 2 explained 15.5% of the variance in the taxa-environment relations (Figure 2). pH, NH_4_-N, and temperature were positively correlated with axis 1 and 2. TP and TN were negatively correlated with axis 1 and positively correlated with axis 2, while DO was negatively correlated with axis 2. *Axis* 1 was therefore interpreted as an environmental gradient of increasing pH, NH_4_-N, and temperature and decreasing TP and TN. *Axis* 2 was interpreted as an environmental gradient of increasing NH_4_-N, TP, and TN and decreasing DO. Taxa with positive scores on axis 1 included Chironomidae, Chlorolestidae, and Leptoceridae, while those with negative scores were Oligochaeta, Syrphidae, Mellanoidae, Caenidae, Baetidae, and Hydropsychidae. Physidae, Erpobdellidae, and Tipulidae had high positive scores on axis 2, while Elmidae, Leptoceridae Acarina, and Leptophlebiidae had negative scores (Figure 2). S2 and S6 were associated with pollution-sensitive species in the EPT taxa such as Leptophlebiidae, Baetidae, Caenidae, Hydropsychidae, and Leptoceridae. S5 and S3 were associated with pollution tolerant taxa such as Physidae, Tipulidae, Cladocera, and Hydrometridae.

### 3.3. Macroinvertebrate Community Composition

A total of 1801 macroinvertebrate individuals belonging to 13 orders and 34 families were collected from the six sites during the sampling period. Family Chironomidae was the most abundant family with 296 individuals, followed by Leptophlebiidae with 181 individuals and Gomphidae with 103 individuals. Orders Hemiptera and Diptera had the highest family representation with four families each with order Diptera having the highest number of individuals at 477. The taxa were made up of one collector-filter (representing 3% of taxa), 9 (or 26%) collector-gatherers, 17 (or 50%) predators, 2 (or 6%) scrappers, and 2 (or 6%) shredders with 3 taxa (Chironomidae, Hydropsychidae and Leptoceridae) having more than two FFGs (Table 3).

The presence/absence of taxa sensitive to pollution among the sample sites was indicative of the site condition based on the SASS and ASPT scores (Table 4). Generally, S6 had the highest SASS scores of more than 100 indicating excellent site condition, while S1 had the lowest SASS score of 41 indicating poor site condition. The sites S6 and S3 had significantly high scores (range 79–113) and ASPT scores (range 4.6–8.1) compared to the other sites (Kruskal–Wallis, *p* < 0.05). For all the sites, the lowest number of taxa was recorded in January and the highest in March.

We did not record Plecoptera in all study sites and therefore was not used in subsequent analysis. Indicator taxa (i.e., % Ephemeroptera and % EPT) significantly differed (Kruskal–Wallis, *p* < 0.05) among the six sampling sites (Table 5). The highest percentage of indicator taxa was recorded at S6, that is, Ephemeroptera (mean: 18.7%), Trichoptera (mean: 6.2%), and EPT (mean: 24.9%), while the lowest percentage of Ephemeroptera (mean: 8.3%) and EPT (mean: 10.6%) was recorded at S1. Trichoptera was the lowest at S5 (1.4%). Diptera had the highest percentage at S1 (mean: 42.7%) and the lowest at S6 (18.4%, Table 5). S1 had a significantly higher mean abundance of collector-gatherers (14.13%) than the other sites (*p* < 0.05). Species richness differed among the sampling sites with higher richness in S6 (mean: 23.3 families) than in the rest of the sites (Kruskal–Wallis, *p*=0.048). Mean diversity indices were not significantly different among the six sampling sites (Kruskal–Wallis, *p* > 0.05, Table 5).

Macroinvertebrate metrics/physicochemical parameters correlations using Pearson are presented in Table 6. EC was significantly positively correlated with % Diptera and Dominance and negatively correlated with %Trichoptera, %EPT, Shannon–Wiener, Margalef Dmg, SASS, and ASPT scores (*r* > 0.50; *p* < 0.01). The nutrients, NH_4_-N, NO_3_-N, and TP were negatively correlated with %Trichoptera (*p* < 0.05). In addition, PO_4_-P and TP were negatively correlated with %EPT, taxa richness, Shannon–Wiener H, and Margalef Dmg (*p* < 0.05).

## 4. Discussion

### 4.1. Physicochemical Water Quality Parameters

Site S1 was the most polluted, while S6 was the least polluted. The difference in water quality status among the sites can be attributed to industrial and domestic activity near the town of Kariba. An increase in organic and inorganic solids and a decrease in DO are physicochemical changes often observed in rivers and streams receiving fish farm effluents [42, 43]. In this study, DO was significantly lower at S5, which receives sources of pollution from a commercial aquaculture factory that has no pretreatment plant, which is located some 200 m from the lake. The aquaculture factory discharges its untreated fat-rich factory effluent straight into the municipal wastewater treatment plant where the fat ends up forming a heavy scum that hinders some treatment processes. Some of the parameters that were high in polluted sites such as nitrates and phosphates indicate pollution by organic waste from residential areas. Our study corroborates with many studies in Zimbabwe where nutrient enrichment has been observed in many water bodies. Phiri et al. [24] found that total nitrates and phosphates were high in polluted sites in Lake Kariba, which is consistent with this study.

Sites S1 and S4 that are located within 500 m of human settlement had high nutrient levels. The frequent breakdown of sewage infrastructure in Kariba because of the lack of proper maintenance due to the current economic situation means raw sewage finds its way into the lake. Nyamhunga, Batonga, and Mahombekombe residential areas are connected to sewage systems and eventually to one wastewater treatment plant, which is in Nyamhunga. Illegal and accidental discharges occur and sewage overflows into the Sanyati basin through the Kasese creek. The creek of sewage passes through the whole Nyamhunga and Batonga areas collecting raw domestic sewage from burst pipes and blocked manholes straight into the Sanyati basin. This has rendered the Sanyati basin organically polluted.

### 4.2. Macroinvertebrate Response to Environmental Variables

Diptera registered the highest number of individuals and families. Dipterans are usually the most abundant and diverse order of insects in freshwaters. This is because they tend to be generalists in their habitat choice inhabiting different environments regardless of the level of degradation [44]. This contrasts with a study by Mezgebu et al. [45] in Ethiopia who found varying abundances of Diptera species across different pollution levels.

Measurement of community structure is usually achieved by calculating diversity indices, which incorporate two aspects: different numbers of taxa (richness) and the distribution of individuals among taxa (evenness). Most diversity indices depend on the quality and availability of habitats [30]. As such, they can reflect the impact of environmental stressors like pollutants independent of ecoregion boundaries. The macroinvertebrate diversity indices in this study failed to delineate the degradation gradient among the sampling sites. Shannon–Wiener's diversity index is sensitive to both relative abundances and the number of species, while Pielou's evenness indices are sensitive to the homogeneity of distribution of abundances among species [46]. Our results are consistent with those of who also found that there was not a clear trend in Shannon–Wiener diversity among the sites sampled in the Upper Mara River Basin in Kenya. On the other hand, taxa richness differed among the sites being generally high in less polluted than more polluted sites. The difference in richness shows that some taxa that are sensitive to pollution may have been eliminated in polluted sites. The decline in taxa richness due to urbanization and industrialization has been reported in other studies in Zimbabwe [10, 18, 47]. In the current study, S1 to S5 where there is human activity within 500 m of the sampled sites had lower species richness compared to S6, which is in a relatively undisturbed site.

Our results showed that the occurrence of certain taxa is closely related to pollution levels and environmental gradients. The percentage of EPT taxa was high in the least polluted site (S6) and lower in the most polluted site (S1). Trichoptera such as Leptoceridae and Hydropsychidae are intolerant to high levels of pollution. In this study, they were associated with S6, which had low organic pollutants. Our study agrees with that of Akamagwuna et al. [48] who found that species of the order Trichoptera are sensitive to organic pollution and are often restricted to undisturbed forests. In our study, S6 has minimal disturbance from human activity as it is located inside a national park. The observation is supported by the SASS and ASPT scores, which were higher in less polluted sites. Except for site S6 that had excellent site condition, the rest of the sites ranged from fair to poor. In their study on the applicability of SASS5 scores in Zimbabwe, Bere & Nyamupingidza [22] found that it generally works in Zimbabwean streams. A high correlation between water parameters and SASS and ASPT scores was observed in the current study, indicating the ability of the scores to detect environmental changes. Despite this agreement, the applicability of the SASS scores still needs to be validated by testing in Zimbabwean reservoirs such as lakes and dams besides rivers and streams.

The results from CCA showed that taxa such as Hydrophilidae and Tipulidae were associated with low organic loads as has been demonstrated in other studies [22, 45]. Diptera (e.g., Syrphidae and Chironomidae) were negatively correlated with DO and positively correlated with nitrates and phosphates. This demonstrates that Diptera taxa tolerate polluted waters. Such results are consistent with other studies on aquatic pollution. For example, Bere and Nyamupingidza [22] found that Syrphidae (Diptera) was associated with nutrient enrichment (ammonium), which is also supported in this study as Syrphidae was associated with total nitrates. In a study around Bulawayo catchment areas, Mwedzi et al. [10] found that Chironomids (Diptera) dominated heavily disturbed areas. This finding supports our study as Chironomids dominated a site that receives domestic and industrial effluent.

Oxygen is a major constraining factor in aquatic insect assemblages with invertebrates utilizing DO facing serious limitations when its levels decrease. However, Hemiptera (e.g., Nepidae and Corixidae) and Diptera (e.g., Culicidae and Syrphidae) have developed air bubbles to increase oxygen supply to their respiratory system, while most species of Chironomidae are capable of surviving oxygen depletion using haemoglobin pigments that help in the transfer of oxygen and the release of oxygen at low external oxygen pressures [49]. In addition, gastropod species in the family Thiaridae are often abundant in polluted waters even at low levels of DO [50]. In this study, the Thiaridae family was associated with S2 and S4, which have organic pollution due to sewage effluent disposal and detergents from washing. The relatively higher abundance of these taxa in low DO waters demonstrates that they can be used as indicators of polluted waters.

Noninsect taxa in the study area, especially Oligochaeta, Atyidae, and Cladocera, responded more strongly to organic pollution as has been observed in other studies [45]. This is in agreement with most other studies where most noninsect taxa increase at degraded sites [51]. Their high abundance at Kasese Bay, a relatively polluted site receiving domestic effluent and raw sewage, indicates that these groups can be used as bioindicators of poor water quality.

The relative abundances of the different FFGs are major characteristics of macroinvertebrate communities with important implications at the ecosystem level and thus can directly relate to community structure and ecosystem functioning [30]. The high number of collector-gatherers in S1 may indicate the presence of high organic matter from littoral vegetation as the study was conducted in the littoral zones of the lake. In addition, collector-gatherers tend to be more generalists in their diet and so tend to be abundant in many aquatic ecosystems and therefore tend to be more tolerant to pollution that might alter the availability of certain foods [52]. The low abundance of predators in this study is expected since specialized feeders are more sensitive and usually represented well in healthy ecosystems.

We recorded low numbers of shredders and no filters. Other studies have also recorded a low number of shredders in the tropics [10, 53]. This is attributed to the fact that microbial action normally replaces shredders in warm tropics and secondary compounds in leaves make them unpalatable [47].

## 5. Conclusions

This study revealed that there is significant pollution on the shoreline of Sanyati Basin, around the town of Kariba especially S1 and S5. Sites S1 and S5 were dominated by the Diptera species especially the family Chironomidae indicating that these sites have high levels of pollution. On the other hand, S6, which is a protected area, was dominated by the taxa of Ephemeroptera and Trichoptera, indicating the low levels of organic pollution in this site. The results support our prediction that physicochemical parameters would affect macroinvertebrate family structure within the study sites, due to the known anthropogenic activities along the shore, and that indicator taxa, that is, Ephemeroptera and Trichoptera, would be associated with areas of low pollution levels and Diptera would be associated with areas of high levels of pollution. The high correlation between water parameters and SASS and ASPT scores was observed in the current study indicating the ability of the scores to detect environmental changes.

## 6. Recommendations

We recommend that water pollutants be regulated at the source. For example, industrial effluents and agricultural pollutants can be controlled at the manufacturer level to reduce their harm. The present study was conducted at the shores of one of the basins of Lake Kariba and with no wider coverage. We suggest that the study be developed to include the pelagic parts of the Lake and the other basins to have a concrete conclusion about the usefulness of macroinvertebrates in delineating the effects of water pollution in Lake Kariba, an important water reservoir for the two nations of Zambia and Zimbabwe. Furthermore, we suggest that an ecotoxicological study be conducted to verify that the presence of the identified families is due to their tolerance to specific pollutants, not accidental.

## Figures and Tables

**Table 1:** Location and description of the study sites.

Site	Coordinates	Activity (within 500 m of the shoreline)
S1	28.8099°E & 16.541°S	Residential area, maintenance of boats and boat activities, and urban and industrial development
S2	28.9503°E & 16.5693°S	Washing and bathing; fishing
S3	29.0414°E & 16.5921°S	Hotels and lodges; road and vehicle activity
S4	28.8959°E & 16.7236°S	Sewage effluent disposal
S5	28.7699°E & 16.8177°S	Commercial aquaculture activity, effluent disposal from farming activities, and urban development
S6	28.6847°E & 16.7894°S	Relatively undisturbed site. Protected area within Matusadona National Park. No human activity within 500 m of the shore zone.

**Table 2:** Mean (±SE) of physicochemical parameters measured at six sites in Sanyati basin (Lake Kariba) for the period January-March 2018.

Parameters	S1	S2	S3	S4	S5	S6	Kruskal-Wallis	
							*H*	*p*
Temperature (°C)	28.6 ± 1.87^a^	28.4 ± 4.08^a^	28.2 ± 1.46^a^	28.1 ± 1.40^a^	28.9 ± 1.88^a^	23.1 ± 0.33^a^	9.67	0.085
pH	7.8 ± 0.16^a^	8.2 ± 0.07^a^	8.1 ± 0.14^a^	8.0 ± 0.09^a^	8.0 ± 0.16^a^	7.3 ± 0.05^b^	11.93	0.036
Conductivity (*μ*Scm^−1^)	125.0 ± 1.14^a^	101.1 ± 1.97^b^	102.5 ± 2.24^b^	103.4 ± 1.16^b^	113.2 ± 4.8^c^	93.44 ± 0.62^d^	17.71	0.003
Turbidity (NTU)	23.6 ± 1.05^a^	20.3 ± 1.38^a^	20.4 ± 1.07^a^	29.9 ± 0.85^a^	25.4 ± 3.92^a^	8.9 ± 0.86^b^	16.72	0.005
DO (mg/L)	3.3 ± 0.31^a^	8.0 ± 0.30^b^	8.1 ± 0.12^b^	7.8 ± 0.26^b^	4.5 ± 0.68^a^	9.4 ± 0.45^b^	19.14	0.002
NH_4_-N (mg/L)	0.15 ± 0.01^a^	0.07 ± 0.01^b^	0.08 ± 0.01^b^	0.10 ± 0.01^b^	0.15 ± 0.02^a^	0.005 ± 0.002^c^	20.93	0.001
NO_3_-N (*µ*/L)	23.2 ± 0.68^a^	16.33 ± 0.85^b^	17.1 ± 0.16^b^	17.9 ± 0.52^b^	25.6 ± 3.36^a^	6.3 ± 0.70^c^	16.45	0.006
TN (mg/L)	0.92 ± 0.06^a^	0.79 ± 0.02^b^	0.54 ± 0.02^c^	0.55 ± 0.01^c^	0.86 ± 0.09^a^	0.49 ± 0.03^c^	17.66	0.003
PO_4_-P (*µ*/L)	29.1 ± 0.49^a^	12.9 ± 0.63^b^	13.4 ± 0.66^b^	12.3 ± 0.52^b^	21.7 ± 3.48^a^	7.0 ± 0.53^c^	17.73	0.003
TP (mg/L)	0.15 ± 0.004^a^	0.08 ± 0.002^b^	0.09 ± 0.004^b^	0.08 ± 0.001^b^	0.10 ± 0.001^b^	0.05 ± 0.003^c^	18.59	0.002

**Table 3:** List of observed macroinvertebrates between January and March 2018 in Sanyati basin, Lake Kariba.

Order	Family	FFG	S1	S2	S3	S4	S5	S6	Total
Acarina	Hydracarina	Predator	0	5	11	6	2	26	50

Cladocera	Cladocera	Scrapper	9	16	9	2	8	18	62

Hirudinea	Erpobdellidae	Predator	12	0	4	2	6	7	31

Oligochaeta	Oligochaeta	Collector-gatherer	26	10	4	0	4	7	51

Neotaenioglossa	Thiaridae	Scrapper	39	10	3	7	5	6	70
	Physidae	Collector-gatherer	0	0	13	5	12	7	37

Decapoda	Atydae	Collector-gatherer	0	3	5	3	0	14	25

Coleoptera	Dytiscidae	Predator	3	8	7	10	2	21	51
	Elmidae	Shredders	0	2	0	3	2	12	19
	Gyrinidae	Predator	0	4	2	6	10	1	23
	Hydrophilidae	Collector-gatherer	12	4	5	3	6	4	34
	Noteridae	Predator	4	7	12	8	9	3	43

Diptera	Chironomidae	Collector-gatherer, predator	89	63	48	40	34	22	296
	Culicidae	Collector-gatherer	36	29	3	4	9	4	85
	Dixidae	Collector-filterer	1	2	0	0	0	1	4
	Empididae	Predator	12	4	6	0	8	9	39
	Syrphidae	Collector-gatherer	11	3	2	3	3	4	26
	Tipulidae	Shredders	12	1	5	3	5	1	27

Ephemeroptera	Baetidae	Collector-gatherer	0	2	3	2	2	12	21
	Caenidae	Collector-gatherer	1	0	2	6	15	44	68
	Leptophlebidae	Collector-gatherer	10	22	27	23	35	64	181

Hemiptera	Belostomatidae	Predator	20	14	3	12	7	1	57
	Corixidae	Predator	6	9	7	9	1	15	47
	Hydrometridae	Predator	0	1	2	1	2	1	7
	Nepidae	Predator	1	2	0	1	1	2	7
	Pleidae	Predator	4	7	12	13	5	3	44
	Veliidae	Predator	1	4	1	6	3	6	21

Megaloptera	Corydalidae	Predator	4	2	6	8	6	4	30

Odonata	Aeshnidae	Predator	3	5	6	1	0	7	22
	Chlorolestidae	Predator	3	2	9	8	6	6	34
	Gomphidae	Predator	29	29	11	17	13	4	103
	Lestidae	Predator	9	14	19	3	13	58	116

Tricoptera	Hydropsychidae	Collector-filterer	3	4	3	9	1	22	42
	Leptoceridae	Shredder, scrapper, predator	0	3	2	1	3	19	28
		Total	360	291	252	225	238	435	1801

**Table 4:** SASS and ASPT index values and number of taxa recorded at six sites in Sanyati basin, Lake Kariba for the period January to March 2018.

Sampling site	Sampling period								
	January			February			March		
	SASS	ASPT	Taxa	SASS	ASPT	Taxa	SASS	ASPT	Taxa
S1	41	4.5	9	58	4.8	12	53	4.4	12
S2	48	6	8	56	5.6	11	66	4.4	15
S3	89	8.1	11	81	6.2	13	79	4.6	17
S4	47	5.2	9	89	5.9	15	75	4.4	17
S5	60	4.6	13	65	5.9	11	71	4.7	15
S6	106	7.6	14	112	6.6	17	113	6.2	18

**Table 5:** Mean (±SE) of macroinvertebrate metrics calculated for six sites in Sanyati basin, Lake Kariba, for the period January-March 2018.

Macroinvertebrate metrics	S1	S2	S3	S4	S5	S6	Kruskal-Wallis	
							*H*	*p*
%Ephemeroptera	8.3 ± 1.45^a^	11.5 ± 2.88^b^	9.3 ± 1.48^a^	12.1 ± 2.05^b^	14.0 ± 2.30^b^	18.7 ± 0.58^c^	11.97	0.035
%Trichoptera	2.3 ± 0.93^a^	2.5 ± 0.90^a^	3.7 ± 1.21^a^	4.1 ± 1.50^a^	1.4 ± 0.68^a^	6.2 ± 0.85^a^	9.03	0.108
%Diptera	42.7 ± 2.62^a^	26.2 ± 2.42^b^	22.2 ± 1.87^b,c^	25.1 ± 2.02^b^	28.5 ± 1.89^b^	18.4 ± 1.24^c^	16.06	0.007
%EPT	10.6 ± 1.33^a^	14.0 ± 3.59^b^	13.1 ± 0.89^b^	16.1 ± 3.09^b^	15.4 ± 1.87^b^	24.9 ± 0.29^c^	12.97	0.024
Collector-gatherers	14.1 ± 4.01^a^	7.1 ± 2.41^b^	4.4 ± 1.28^c^	3.8 ± 0.80^c^	6.3 ± 1.85^b^	6.3 ± 1.71^b^	12.02	0.021
Predators	6.6 ± 1.92^a^	6.9 ± 1.70^a^	6.9 ± 1.20^a^	6.5 ± 1.17^a^	5.5 ± 0.99^a^	10.2 ± 3.45^a^	1.58	0.891
Shredders	6.1 ± 0.71^a^	3.0 ± 1.01^a^	2.5 ± 0.21^a^	4.0 ± 0.92^a^	5.3 ± 1.23^a^	6.0 ± 3.10^a^	1.98	0.856
Taxa richness	15.5 ± 1.66^a^	15.8 ± 1.70^a^	18.3 ± 1.32^a^	16.5 ± 1.32^a^	17.3 ± 2.2^a^	23.3 ± 1.44^b^	10.70	0.048
Dominance (D)	0.14 ± 0.02^a^	0.09 ± 0.004^a^	0.08 ± 0.005^a^	0.09 ± 0.009^a^	0.09 ± 0.01^a^	0.06 ± 0.003^a^	10.84	0.052
Shannon-Wiener (*H*)	2.3 ± 0.12^a^	2.5 ± 0.07^a^	2.7 ± 0.06^a^	2.6 ± 0.09^a^	2.6 ± 0.13^a^	3.0 ± 0.05^a^	5.04	0.232
Pielou evenness (*J*)	0.66 ± 0.03^a^	0.82 ± 0.03^a^	0.83 ± 0.02^a^	0.83 ± 0.03^a^	0.77 ± 0.01^a^	0.76 ± 0.02^a^	8.96	0.16
Margalef (Dmg)	3.3 ± 0.33^a^	3.4 ± 0.39^a^	3.8 ± 0.28^a^	3.6 ± 0.21^a^	3.7 ± 0.45^a^	4.7 ± 0.24^a^	8.93	0.112

**Table 6:** Spearman rank correlation coefficients (*r*) between physicochemical variables and macroinvertebrate metrics in Sanyati Basin, with significant correlations highlighted as *p* < 0.05 (^*∗*^) and *p* < 0.01 (^*∗∗*^).

	Temperature (°C)	pH	Conductivity (*μ*Scm^−1^)	Turbidity (NTU)	DO (mg/L)	NH_4_-N (mg/L)	NO_3_-N (*µ*/L)	TN (mg/L)	PO_4_-P (*µ*/L)	TP (mg/L)
%Ephemeroptera	−0.14	−0.37	−0.31	−0.15	0.31	−0.30	−0.34	−0.27	−0.41^*∗*^	−0.37
%Trichoptera	−0.19	−0.46^*∗*^	−0.57^*∗*^	−0.36	0.35	−0.45^*∗*^	−0.47^*∗*^	−0.38	−0.40	−0.48^*∗*^
%Diptera	0.50^*∗*^	0.17	0.76^*∗∗*^	0.45	−0.82^*∗∗*^	0.70^*∗∗*^	0.60^*∗∗*^	0.71^*∗∗*^	0.78^*∗∗*^	0.73^*∗∗*^
%EPT	−0.18	−0.10	−0.60^*∗*^	−0.23	0.39	−0.41^*∗*^	−0.44^*∗*^	−0.32	−0.48^*∗*^	−0.52^*∗∗*^
Taxa richness	−0.70^*∗∗*^	0.04	−0.58^*∗∗*^	−0.48^*∗*^	0.57^*∗∗*^	−0.47^*∗*^	−0.42^*∗*^	−0.39	−0.55^*∗∗*^	0.55^*∗∗*^
Dominance (D)	0.51^*∗*^	−0.04	0.70^*∗∗*^	0.47^*∗*^	−0.69^*∗∗*^	0.63^*∗∗*^	0.62^*∗∗*^	0.59^*∗∗*^	0.70^*∗∗*^	−0.69^*∗∗*^
Shannon-Wiener (H)	−0.60^*∗∗*^	0.32	−0.67^*∗∗*^	−0.45^*∗*^	0.67^*∗∗*^	−0.56^*∗∗*^	−0.54^*∗∗*^	−0.54^*∗∗*^	−0.67^*∗∗*^	−0.67^*∗∗*^
Pielou evenness (*J*)	0.27	0.32	−0.21	0.14	0.25	−0.26	−0.31	−0.47^*∗*^	−0.29	−0.17
Margalef (Dmg)	−0.68^*∗∗*^	−0.05	−0.57^*∗∗*^	−0.47^*∗*^	0.55^*∗∗*^	−0.46^*∗*^	−0.39	−0.033	−0.54^*∗∗*^	−0.57^*∗∗*^
SASS	0.34	0.79^*∗∗*^	−0.67^*∗∗*^	−0.64^*∗∗*^	0.87^*∗∗*^	−0.56^*∗*^	−0.61^*∗∗*^	−0.62^*∗∗*^	−0.56^*∗∗*^	−0.58^*∗∗*^
ASPT	0.33	0.79^*∗∗*^	−0.66^*∗∗*^	−0.67^*∗∗*^	0.84^*∗∗*^	−0.55^*∗*^	−0.63^*∗∗*^	−0.66^*∗∗*^	−0.52^*∗∗*^	−0.56^*∗∗*^

## Data Availability

The data used to support the findings of this study are available from the corresponding author upon reasonable request.
